# Supplementary material for: In silico repositioning of approved drugs against Schistosoma mansoni energy metabolism targets
Source: PLoS One. 2018 Dec 31;13(12):e0203340. doi: 10.1371/journal.pone.0203340 (PMC6312253; doi:10.1371/journal.pone.0203340)
Supplement: S1 Fig — (CP1A2_HUMAN): Cytochrome P450 1A2 (CP1A1_HUMAN): Cytochrome P450 1A1. (PDF) [file pone.0203340.s001.pdf]

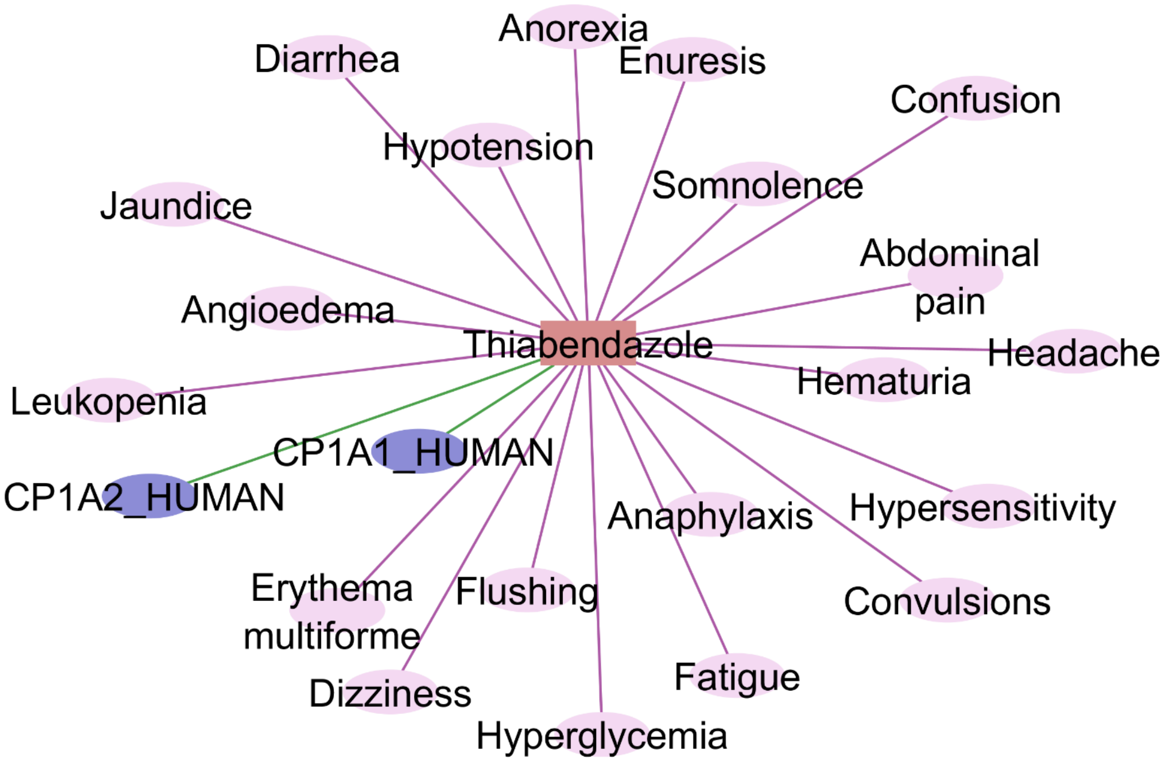

S1 Figure. Network of interaction between the drug thiabendazole and targets of human metabolism, as well as the relationship with the side effects.
