# Supplementary material for: In silico repositioning of approved drugs against Schistosoma mansoni energy metabolism targets
Source: PLoS One. 2018 Dec 31;13(12):e0203340. doi: 10.1371/journal.pone.0203340 (PMC6312253; doi:10.1371/journal.pone.0203340)
Supplement: S2 Fig — (CP3A4_HUMAN): Cytochrome P450 3A4, (CP1A2_HUMAN): Cytochrome P450 1A2, (CP1A1_HUMAN): Cytochrome P450 1A1. (PDF) [file pone.0203340.s002.pdf]

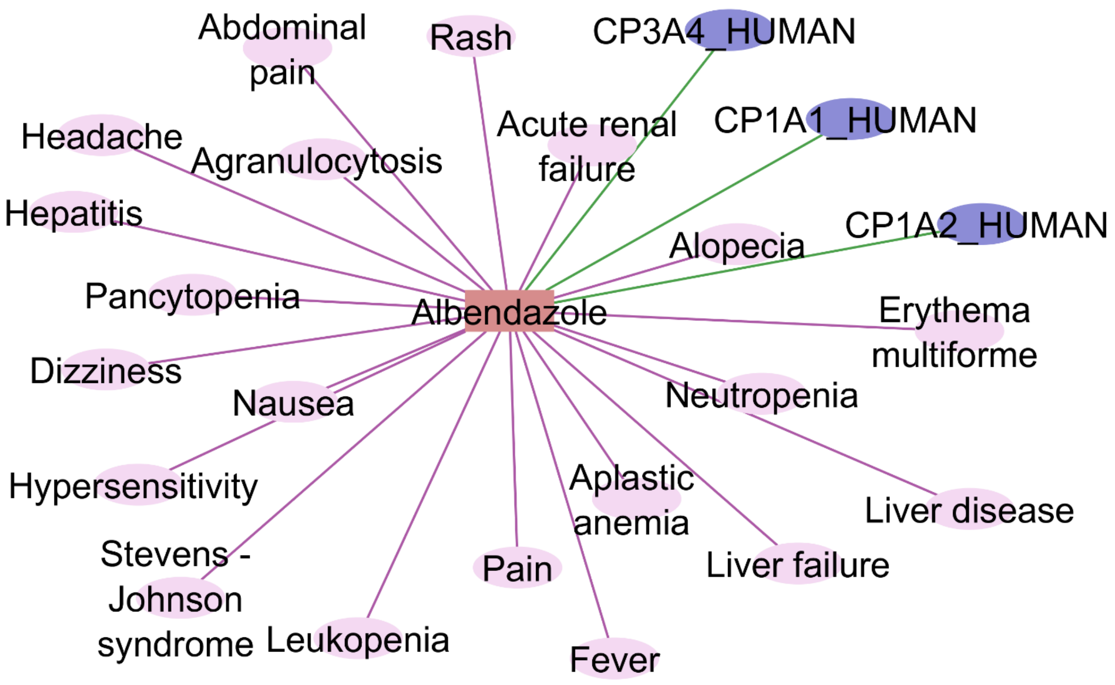

S2 Figure. Network of interaction between albendazole and protein targets of human metabolism, as well as the relationship with side effects
