# Supplementary material for: In silico repositioning of approved drugs against Schistosoma mansoni energy metabolism targets
Source: PLoS One. 2018 Dec 31;13(12):e0203340. doi: 10.1371/journal.pone.0203340 (PMC6312253; doi:10.1371/journal.pone.0203340)
Supplement: S3 Fig — (ADH1A_HUMAN): Alcohol dehydrogenase 1A, (ADH1B_HUMAN): Alcohol dehydrogenase 1B, (ADH1G_HUMAN): Alcohol dehydrogenase 1C, (CATA_HUMAN): Catalase, (CP2A6_HUMAN): Cytochrome P450 2A6, (CP2E1_HUMAN): Cytochrome P450 2E1. (PDF) [file pone.0203340.s003.pdf]

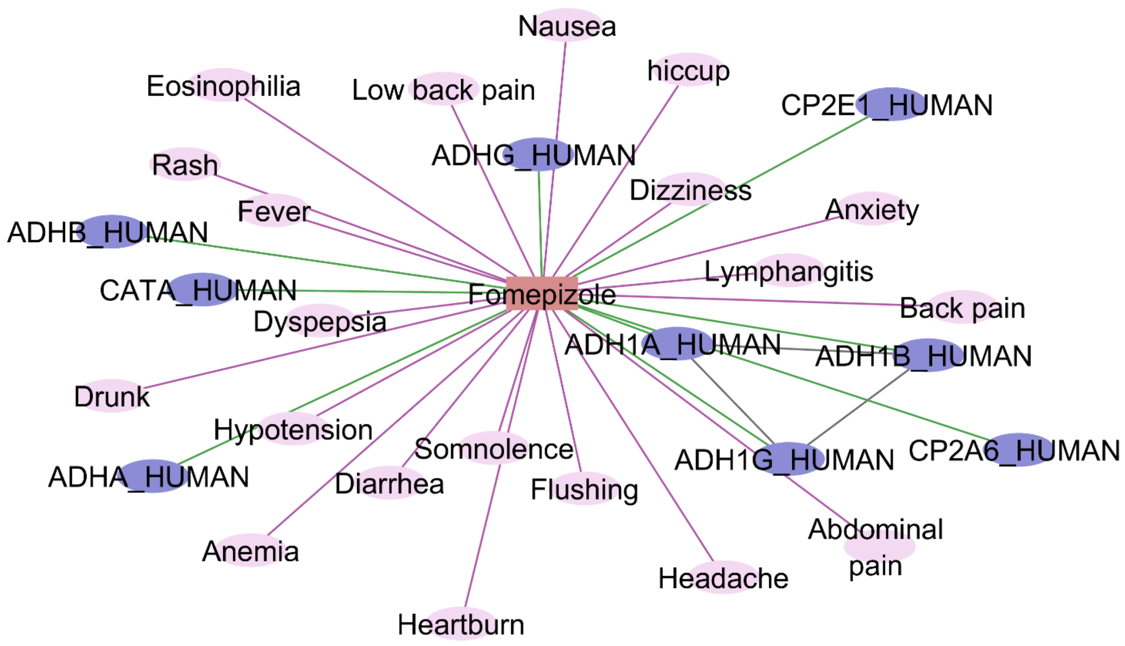

S3 Figure. Network of interaction between fomepizole and protein targets of human metabolism, as well as the relationship with side effects.
