# Supplementary material for: In silico repositioning of approved drugs against Schistosoma mansoni energy metabolism targets
Source: PLoS One. 2018 Dec 31;13(12):e0203340. doi: 10.1371/journal.pone.0203340 (PMC6312253; doi:10.1371/journal.pone.0203340)
Supplement: S4 Fig — (GRIA1_HUMAN): Glutamate receptor 1, (GLRA1_HUMAN): Glycine receptor alpha-1 subunit, (ATPD_HUMAN): ATP delta synthase subunit, mitochondrial, (GBRA1_HUMAN): Gamma-aminobutyric acid receptor alpha-1 subunit, (AT2C1_HUMAN): ATPase for calcium transport type 2C member 1, (KCNA1_HUMAN): Potassium voltage-gated channel subfamily A member 1, (NU1M_HUMAN): NADH-ubiquinone oxidoreductase. (PDF) [file pone.0203340.s004.pdf]

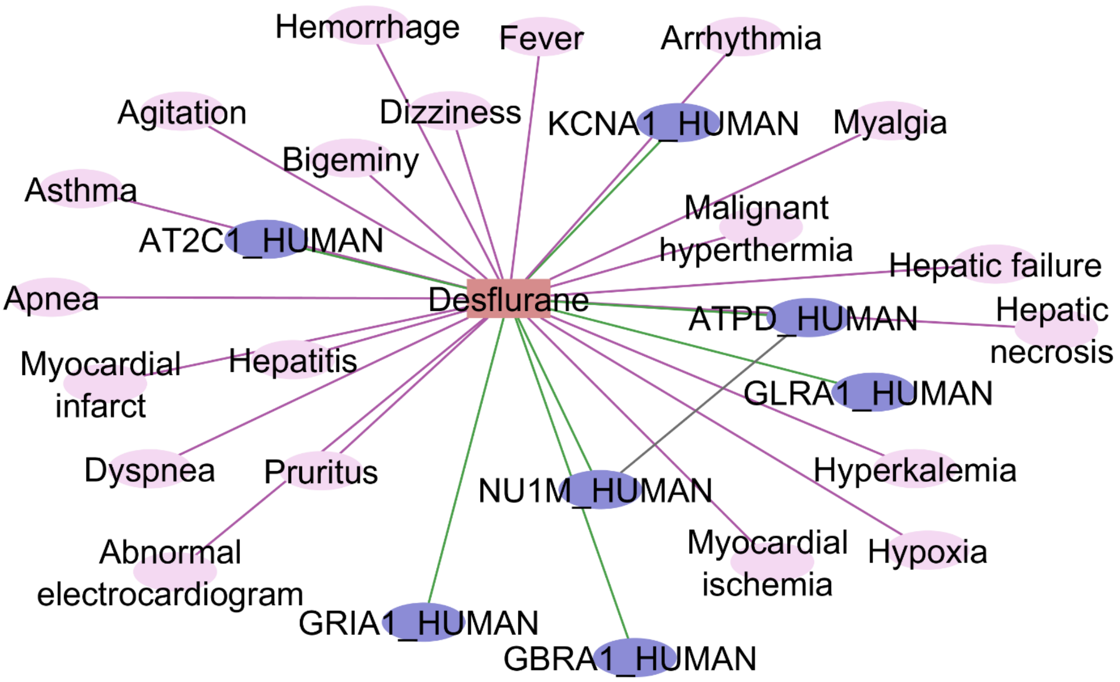

S4 Figure. Network of interaction between desflurane and protein targets active in the metabolism of the human body, as well as the relationship with side effects.
