# Supplementary material for: In silico repositioning of approved drugs against Schistosoma mansoni energy metabolism targets
Source: PLoS One. 2018 Dec 31;13(12):e0203340. doi: 10.1371/journal.pone.0203340 (PMC6312253; doi:10.1371/journal.pone.0203340)
Supplement: S5 Fig — (GLRA1_HUMAN): Glycine alpha-1 receptor subunit, (CP2B6_HUMAN): Cytochrome P450 2B6, (CALM HUMAN): Calmodulin, (GRIA3_HUMAN): Glutamate 3 receptor, (GRIA1_HUMAN): Glutamate 1 receptor, (NU1M_HUMAN): NADH-ubiquinone oxidoreductase, (AT2C1_HUMAN): ATPase for calcium transport type 2C member 1, (ATPD_HUMAN): ATP delta synthase subunit, mitochondrial, (CP2E1_HUMAN): Cytochrome P450 2E1, (KCNA1_HUMAN): Potassium voltage-gated channel for subfamily 1 member 1, (GBRA1_HUMAN): Gamma-aminobutyric acid receptor alpha-1 subunit. (PDF) [file pone.0203340.s005.pdf]

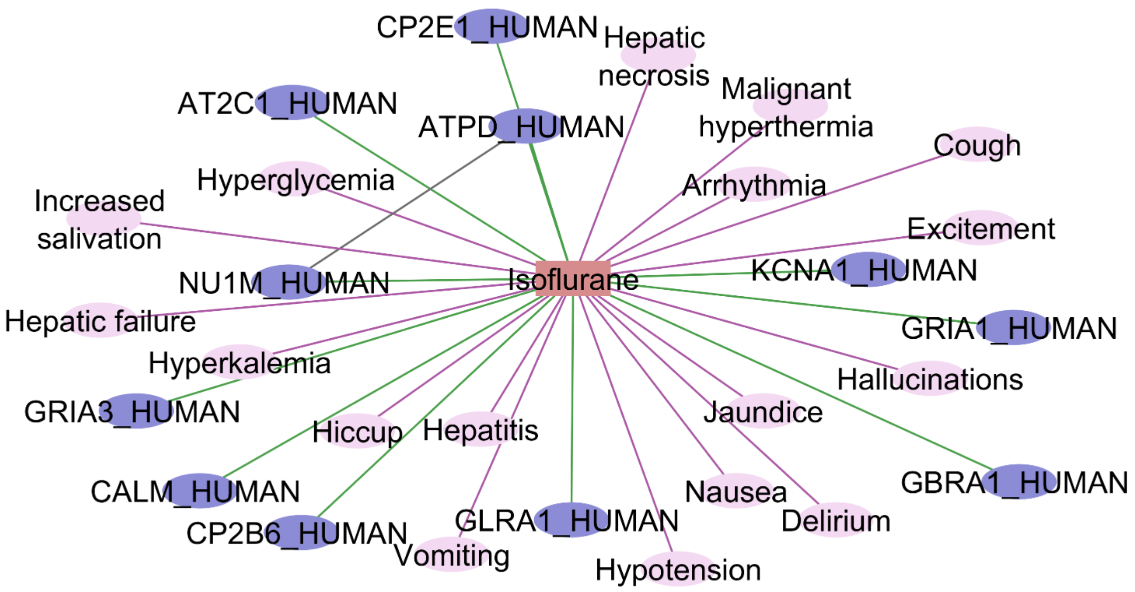

S5 Figure. Network of interaction between isoflurane and protein targets active in the metabolism of the human body, as well as the relationship with side effects.
