# Supplementary material for: In silico repositioning of approved drugs against Schistosoma mansoni energy metabolism targets
Source: PLoS One. 2018 Dec 31;13(12):e0203340. doi: 10.1371/journal.pone.0203340 (PMC6312253; doi:10.1371/journal.pone.0203340)
Supplement: S6 Fig — (PTN4_HUMAN): Tyrosine phosphatase type 4, (FPPS_HUMAN): Farnesyl pyrophosphate synthase. (PDF) [file pone.0203340.s006.pdf]

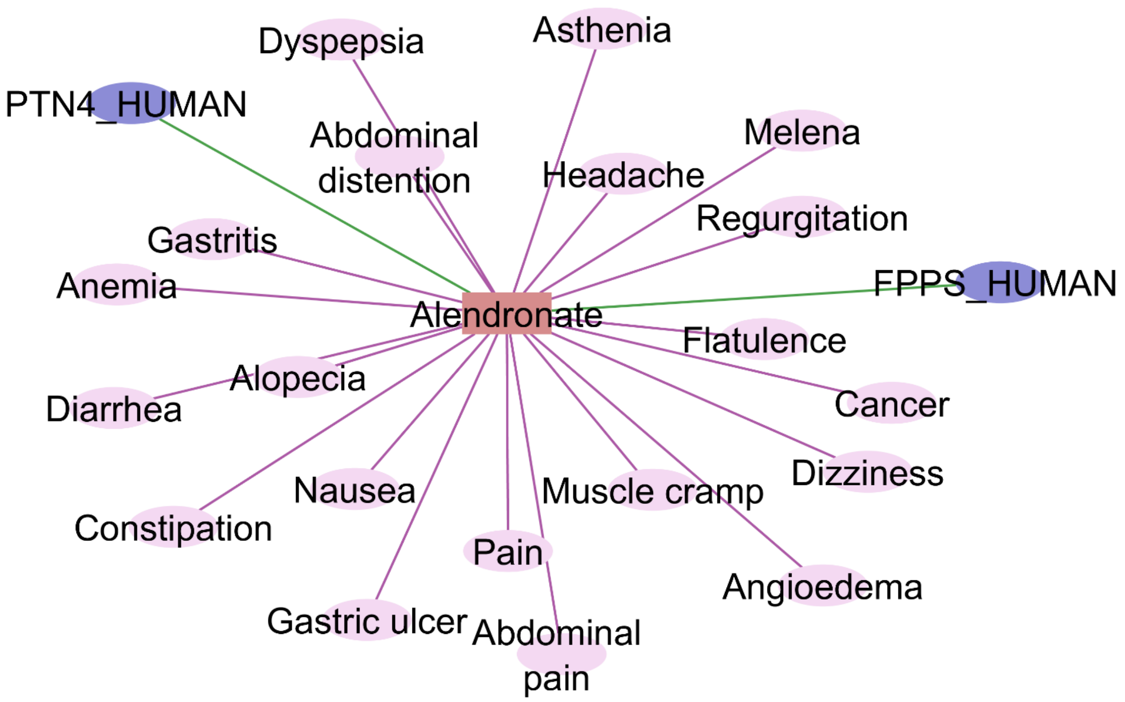

S6 Figure. Network of interactions between Alendronate and protein targets active in the metabolism of the human body, as well as the relationship with the side effects
