# Supplementary material for: In silico repositioning of approved drugs against Schistosoma mansoni energy metabolism targets
Source: PLoS One. 2018 Dec 31;13(12):e0203340. doi: 10.1371/journal.pone.0203340 (PMC6312253; doi:10.1371/journal.pone.0203340)
Supplement: S7 Fig — (ABCC8_HUMAN): ATP-binding cassette subfamily C member 8, (AT1A1_HUMAN): ATPase transport of sodium/potassium alpha 1 subunit, (IRK11_HUMAN): ATP-sensitive inward rectifier potassium channel 11, (KCMA1_HUMAN): Calcium-activated potassium channel subunit alpha-1, (Q59GM5_HUMAN): Sulfonylurea receptor, (CAH1_HUMAN): carbonic anhydrase 1, (CAH2_HUMAN): carbonic anhydrase 2, (CAH4_HUMAN): carbonic anhydrase 4, (S12A3_HUMAN): solute carrier family 12 member 3. (PDF) [file pone.0203340.s007.pdf]

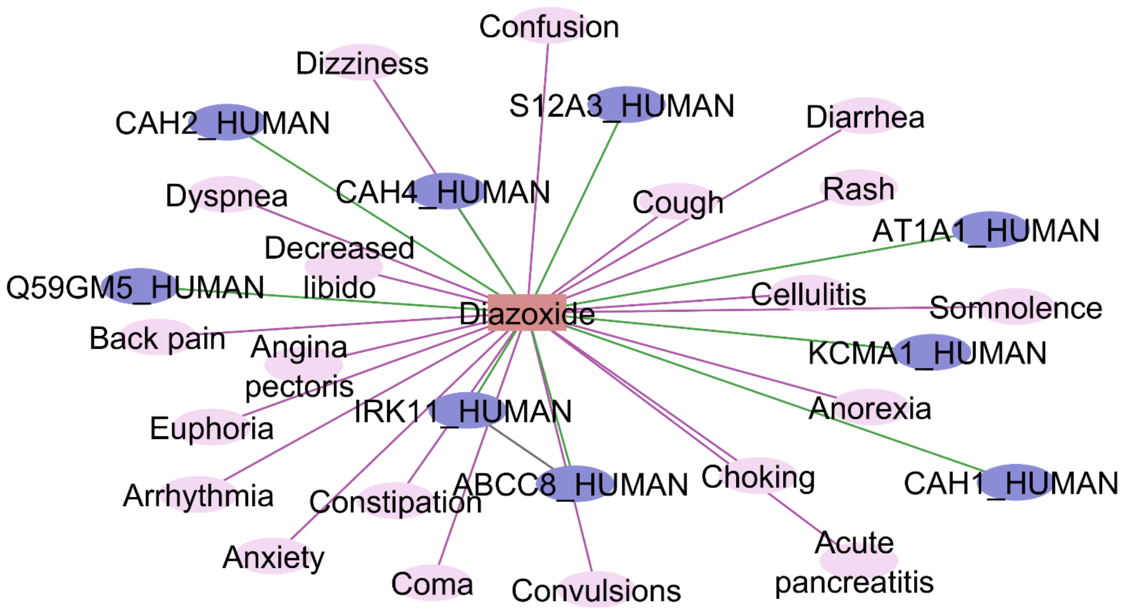

S7 Figure. Network of interactions between diazoxide and protein targets active in the metabolism of the human body, as well as the relationship with side effects.
